# Pregnancy outcomes amongst thalassemia traits

**DOI:** 10.1007/s00404-013-2886-9

**Published:** 2013-05-17

**Authors:** Tharangrut Hanprasertpong, Ounjai Kor-anantakul, Roengsak Leetanaporn, Thitima Suntharasaj, Chitkasaem Suwanrath, Ninlapa Pruksanusak, Savitree Pranpanus

**Affiliations:** Department of Obstetrics and Gynecology, Faculty of Medicine Prince of Songkla University, Hat Yai, Songkhla, 90110 Thailand

**Keywords:** Thalassemia trait, Pregnancy outcome, Normal

## Abstract

**Objective:**

To compare the pregnancy outcome between pregnancies affected and not affected by thalassemia trait.

**Methods:**

A retrospective case–control cohort study was conducted on singleton pregnant women who attended antenatal care and delivered at Songklanagarind Hospital. All of the participating thalassemia trait pregnant women were diagnosed based on hemoglobin typing and/or DNA analysis. A ratio of around 1–1 was used to compare their pregnancy outcomes with normal pregnant women.

**Results:**

Seven hundred thirty-nine thalassemia trait and 799 normal pregnant women were included in the study. All of the women were Thai nationals living in the Southern Region of Thailand and nearly all of them had spontaneously conceived. Maternal complication rates of gestational diabetes, preterm birth, antepartum bleeding, postpartum bleeding, shoulder dystocia and puerperal morbidity, and the rates of neonatal complications: macrosomia, fetal weight <2,000 g, intrauterine growth restriction (IUGR), stillbirth, low Apgar score (<7) at 1 and 5 min and NICU admission, were not significantly different between the two groups. The rate of pre-eclampsia, however, was significantly different, with RRs of 1.73 (CI 1.01–3.00).

**Conclusion:**

The thalassemia trait condition did not affect the risk of gestational diabetes, postpartum hemorrhage, stillbirth, preterm birth and puerperal morbidity. However, pre-eclampsia should be warranted especially among nulliparous and high-BMI pregnant women.

## Introduction

A normal human hemoglobin molecule arranges in a tetramer which consists of a central haem surrounded by four globin chains. The hemoglobin constituents from the embryonic to the adult period vary, starting with two zeta and two epsilon subunits during the embryonic stage. Then, from 6 to 7 weeks of gestation to a few months after birth, it consists of to two alpha and two gamma subunits which is called fetal hemoglobin. Lastly, there are two forms of hemoglobin during adulthood. The first is called hemoglobin A (Hb A), which consists of two alpha and two beta subunits and the second is hemoglobin A2 (Hb A2), which accounts for <3.5 % of all adult hemoglobin. Hb A2 consists of two alpha and two delta subunits [1].

Thalassemia is an autosomal recessive disorder which affects the hemoglobin synthesis process. Thalassemia types vary according to which gene of the globin molecule is affected. The major types are the α and β thalassemia. Furthermore, a single point mutation in the beta gene results in an abnormal hemoglobin called hemoglobin E (Hb E). Thalassemia trait is a genetic trait and not a health disease; it dose not progress into the more serious forms of thalassemia, which need medical treatment. Alpha-thalassemia-1 (α-1) trait describes the situation when two alleles on the same chromosome are deleted. Beta-thalassemia (β) and E-thalassemia traits denote one allele mutation and one point mutation in the globin allele, respectively. Usually, the α-thalassemia-1, β-thalassemia and E thalassemia traits present with mild microcytic hypochromic anemia without clinical symptoms. The prevalence of thalassemia trait in Thai pregnant women is high. The overall prevalence has been reported 25.4 % and categorized as follow: 6.6 % for the α-1 trait, 3.7 % for the β trait, 11.6 % for the E trait, 0.8 % for the homozygous E, 1.2 % for the combined α-1 and β trait, and 1.5 % for the combined α-1 and E trait [2].

Regarding the thalassemia disease in women, physiologic changes during pregnancy worsen the severity of anemia and are significantly associated with an increased risks of fetal growth restriction, preterm birth and low birthweight [3–5]. There are some reports on pregnancy outcomes among women with thalassemia trait [6, 7], but, to our knowledge, no report on the risk of adverse pregnancy outcomes when compared with pregnant women without thalassemia trait has been published. The objective of the present study was to determine the risk for adverse pregnancy outcomes in pregnancies complicated by thalassemia trait.

## Materials and methods

This retrospective, case-controlled, cohort study was conducted at the Maternal Fetal Medicine Unit, Department of Obstetrics and Gynecology, Songklanagarind Hospital using its thalassemia database of the Department of Obstetrics and Gynecology and the computer database of patient records of the Antenatal Clinic, labor records and those of the Obstetric Inpatient Ward. Our hospital’s computer database has been established since January 2007. The inclusion criteria for the study group were the following: (1) β trait and E trait diagnosed during or before pregnancy by hemoglobin typing, (2) α-1 trait diagnosed during or before pregnancy by DNA analysis, (3) singleton pregnancy with antenatal care and delivery at Songklanagarind Hospital, (4) available data for pregnancy outcome, and (5) no other medical or surgical complications during pregnancy. The control group participants were recruited from the general obstetrics database in the same period using a controls-to-cases ratio of around 1:1. The inclusion criteria for the control group were the following: (1) singleton pregnancy with no medical or surgical complication, (2) antenatal care and delivery at Songklanagarind Hospital, and (3) available data for pregnancy outcome. The exclusion criteria for both groups were; (1) diagnosed fetal structural or chromosomal abnormalities during pregnancy, and (2) spontaneous or induced abortion before 24 weeks of gestation. This study was approved by our institution’s Ethics committee.

Gestational age was determined at the first visit, either by last menstrual period, pelvic examination or ultrasound. In accordance with our hospital’s policy, only the pregnant women who presented for a first antenatal care visit during the first trimester, were checked for thalassemia because the limitations of termination mean that termination is available only before 24 weeks of gestation. All of the participating pregnant women were regularly scheduled for antenatal care by obstetricians at Songklanagarind Hospital. They underwent a complete blood count, urinary analysis, hepatitis B, and HIV infection as well as cervical cancer screening at the first visit. On followed-up visits, they were evaluated for blood pressure, weight gain, fundal height, fetal heart rate and urine tests for proteinuria and glucosuria. Additionally, ultrasonography was performed as per maternal or fetal indication.

The maternal records along with pregnancy and neonatal outcomes were reviewed. Maternal record data of interest included age, parity, body mass index, educational level, smoking, history of assisted reproductive techniques, red blood cell indices profile and obstetric complications such as pre-eclampsia, gestational diabetes, preterm labor/-birth, antenatal bleeding, postpartum hemorrhage, puerperal morbidity, heart failure and thromboembolism. Pregnancy and neonatal outcomes of interest were the following: (1) preterm birth, defined as a live birth before 37 weeks of gestation, (2) fetal intrauterine growth restriction (IUGR), defined as a birth weight <10 % of the normal growth curve, (3) macrosomia, defined as a birth weight more than the 90 % of the normal growth curve, (4) stillbirth, defined as death in utero after 24 weeks of gestation, (5) Apgar scores at 1 and 5 min, and (6) intrapartum complications such as shoulder dystocia.

The statistical analysis was performed with the R 2.10.0 software (freeware distributed by the R development core team). The descriptive data are presented as percentage or mean and standard deviation. Statistical significance was determined using the *χ* ^2^ and the *t* test for differences in qualitative and continuous variables, respectively. Difference in pregnancy outcome between both groups is shown as relative risk and the 95 % confidence intervals (CIs) were computed. A *P* < 0.05 was considered statistically significant.

## Results

There were 739 and 799 women in the study and control groups, respectively. All of them were Thai nationals living in the Southern Regions of Thailand. Nearly half of the women in both groups were individual employee (47.3 and 43.9 % in study and control groups, respectively). Only two women smoked, and both of them had thalassemia trait. Nearly all of the investigated women spontaneously conceived, two of them had a history of ovulation induction. Both groups were comparable in terms of maternal age and educational level, but parity, body mass index (BMI) and the mean Hemoglobin (Hb) level were statistically different. The number of nulliparous women in the study group was greater than that in the control group. Moreover, the mean BMI in the study group was higher than that of the control group (27.47 ± 3.63 vs. 26.62 ± 4.33 kg/m^2^) and mean Hb level in the study group was below that of the control group (11.27 ± 1.08 vs. 12.35 ± 1.03 g/dl). No fetal malformation was either prenatally or postnatally diagnosed in our study.

The mean gestational ages at birth were not significantly different between the study and control groups (38.35 ± 1.73 vs. 38.42 ± 1.67; *P* = 0.21). The differences in the rates of maternal complication, which included gestational diabetes, preterm birth, antepartum bleeding, postpartum bleeding, shoulder dystocia and puerperal morbidity, and the rates of neonatal complication, which included macrosomia, fetal weight <2,000 g, intrauterine growth restriction (IUGR), stillbirth, low Apgar score (<7) at 1 and 5 min and NICU admission, were not significantly different. The rate of pre-eclampsia was slightly higher in the study group than that in the control group. However, the difference was significantly different [RR (95 % CI) 1.73 (1.01–3.00)] (Tables 1, 2). There were no heart failure and thromboembolism cases reported in our study.

**Table 1:** Baseline characteristics of pregnant women with thalassemia trait and controls

Characteristics	Study group (*n* = 739)	Control group (*n* = 799)	*P* value
Maternal age (years)			
≤18	11	5	0.115
18–35	608	611	
≥35	136	163	
Parity			
Nulliparous	445 (60.2)^a^	392 (49.1)	0.001
Parous	294 (39.8)	407 (50.9)	
Mean BMI (kg/m^2^)	27.47 ± 3.63	26.62 ± 4.33	0.000
Hb level (g/dl)	11.27 ± 1.08	12.35 ± 1.03	0.000
Primary education or less	350 (47.4)^a^	385 (48.2)	0.30

^a^Values are given as number (%)

**Table 2:** Pregnancy outcomes in both groups

Outcome	Study group *n* (%) (*n* = 739)	Control group *n* (%) (*n* = 799)	Relative risk (95 % CI)
Gestational diabetes	31 (4.2)	35 (4.4)	0.93 (0.58–1.50)
Pre-eclampsia	33 (4.5)	20 (2.5)	1.73 (1.01–3.00)
Antepartum hemorrhage	7 (0.9)	4 (0.5)	1.84 (0.54–6.28)
Postpartum hemorrhage	22 (3.0)	23 (2.9)	1.02 (0.57–1.81)
Preterm birth	49 (6.6)	44 (5.5)	1.17 (0.79–1.74)
Macrosomia	37 (5.0)	58 (7.3)	0.67 (0.45–1.00)
IUGR	13 (1.8)	13 (1.6)	1.05 (0.49–2.26)
Fetal weight <2,500 g	44 (6.0)	59 (7.4)	0.77 (0.53–1.12)
Stillbirth	4 (0.5)	2 (0.3)	2.14 (0.39–11.67)
Apgar score ≤7 at 1 min	35 (4.7)	46 (5.8)	0.82 (0.53–1.25)
Apgar score ≤7 at 5 min	7 (0.9)	5 (0.6)	1.50 (0.48–4.71)
NICU admission	12 (1.6)	12 (1.5)	1.06 (0.48–2.35)
Shoulder dystocia	0 (0)	2 (0.3)	000
Puerperal morbidity	8 (1.1)	5 (0.6)	1.70 (0.56–5.17)

## Discussion

To the best of our knowledge, this is the first case- controlled, cohort study comparing the pregnancy outcomes between pregnancies affected and not affected by thalassemia trait. Although two previous studies on pregnancies affected by thalassemia trait have been reported, one of them focused only on gestational diabetes [6] and the other included haemoglobinopathy carries which combined sickle cell trait and thalassemia trait [7]. In our study, the rates of common obstetric complication, such as gestational diabetes, preterm birth, antepartum hemorrhage, postpartum hemorrhage, macrosomia, IUGR, fetal weight <2,500 g, stillbirth, Apgar score equal or <7 at 1 and 5 min, NICU admission, shoulder dystocia and puerperal morbidity, were comparable. Concerning the significant differences in parity and BMI between the control and study groups that may affect the interpretation of results, a higher number of nulliparous women and a higher mean BMI in the study groups were observed. The association of adverse pregnancy outcome and maternal obesity has been reported in several studies [8–10]. The adverse pregnancy outcomes include gestational diabetes, pre-eclampsia, postpartum hemorrhage, stillbirth, preterm birth and puerperal morbidity [8–10]. However, the magnitude of adverse pregnancy outcome increases with higher BMI values [8]. In our study, the mean BMI in both groups fell in the overweight range, but it was higher in the study group. Nevertheless, most adverse pregnancy outcomes in both groups, except pre-eclampsia, were similar. Our finding that most adverse pregnancy outcomes in pregnant women affected and not affected by thalassemia trait were not significantly different contradicts that of Lao et al. reporting that the presence of the α-thalassemia trait is an additional risk factor for gestational diabetes mellitus. This difference in gestational diabetic outcome may be explained by the larger number of participants and inclusion criteria of our study, which included more types of thalassemia trait.

A higher incidence of pre-eclampsia in the thalassemia group was found in our study, but its relative risk was slightly above one. Moreover, the large number of nulliparous and higher BMI women in study groups may have affected the pre-eclampsia outcome. The association between high BMI and nulliparous status with pre-eclampsia has already been described [8–11]. Nonetheless, Maruotti et al. found that the rate of pre-eclampsia in pregnant women affected by β-thalassemia and who had undergone chorionic villus sampling (CVS) were significantly lower than those of pregnant women who were not performed CVS. They hypothesized that a lower maternal hemoglobin level is a protective mechanism for pre-eclampsia. An increasing in trophoblast invasion into the spiral arteries accompanied by an unchanged apoptosis leads to spiral artery lumen widening [12]. Hence, the pre-eclampsia outcome in pregnant women affected by thalassemia trait should be further investigated.

Our findings warrant the conclusion that the thalassemia trait condition dose not affect the risk of gestational diabetes, postpartum hemorrhage, stillbirth, preterm birth and puerperal morbidity. However, the risk for pre-eclampsia is heightened in such individuals, especially among nulliparous and high-BMI pregnant women.
